# Completeness of Maternal Smoking Status Recording during Pregnancy in United Kingdom Primary Care Data

**DOI:** 10.1371/journal.pone.0072218

**Published:** 2013-09-19

**Authors:** Nafeesa N. Dhalwani, Laila J. Tata, Tim Coleman, Kate M. Fleming, Lisa Szatkowski

**Affiliations:** 1 Division of Epidemiology and Public Health, University of Nottingham, Nottingham, United Kingdom; 2 Division of Primary Care, University of Nottingham, Nottingham, United Kingdom; The Ohio State University, United States of America

## Abstract

**Background:**

Given the health impacts of smoking during pregnancy and the opportunity for primary healthcare teams to encourage pregnant smokers to quit, our primary aim was to assess the completeness of gestational smoking status recording in primary care data and investigate whether completeness varied with women's characteristics. As a secondary aim we assessed whether completeness of recording varied before and after the introduction of the Quality and Outcomes Framework (QOF).

**Methods:**

In The Health Improvement Network (THIN) database we calculated the proportion of pregnancies ending in live births or stillbirths where there was a recording of maternal smoking status for each year from 2000 to 2009. Logistic regression was used to assess variation in the completeness of maternal smoking recording by maternal characteristics, before and after the introduction of QOF.

**Results:**

Women had a record of smoking status during the gestational period in 28% of the 277,552 pregnancies identified. In 2000, smoking status was recorded in 9% of pregnancies, rising to 43% in 2009. Pregnant women from the most deprived group were 17% more likely to have their smoking status recorded than pregnant women from the least deprived group before QOF implementation (OR 1.17, 95% CI 1.10–1.25) and 42% more likely afterwards (OR 1.42, 95% CI 1.37–1.47). A diagnosis of asthma was related to recording of smoking status during pregnancy in both the pre-QOF (OR 1.63, 95% CI 1.53–1.74) and post-QOF periods (OR 2.08, 95% CI 2.02–2.15). There was no association between having a diagnosis of diabetes and recording of smoking status during pregnancy pre-QOF however, post-QOF diagnosis of diabetes was associated with a 12% increase in recording of smoking status (OR 1.12, 95% CI 1.05–1.19).

**Conclusion:**

Recording of smoking status during pregnancy in primary care data is incomplete though has improved over time, especially after the implementation of the QOF, and varies by maternal characteristics and QOF-incentivised morbidities.

## Introduction

Smoking during pregnancy has a well-documented negative effect on the health of a mother and her baby [1] and smoking cessation during pregnancy has been linked to a reduction in maternal and fetal complications in addition to its wider health benefits [2], [3]. Current recommendations emphasise that all healthcare workers involved in a pregnant woman's care (e.g. midwives, general practitioners (GPs), practice nurses and obstetricians) should assess the woman's smoking status at the earliest possible stage of pregnancy and offer cessation advice and a referral to specialist stop smoking advisers for women who smoke [4]–[8]. Documentation of a woman's smoking status in her medical records is recommended to enable her healthcare team to offer appropriate support throughout the pregnancy [7].

In the United Kingdom (UK) women must be registered with a GP in order to receive antenatal care and, although most antenatal contacts are with midwives, an estimated 77% of women see their GPs first for confirmation of pregnancy before attending an antenatal booking appointment with a midwife [9]. This first contact with a GP and subsequent visits during pregnancy could potentially be used as an opportunity for assessing and recording the smoking status of pregnant women.

In April 2004, a new contract for GPs was implemented which introduced a number of pay-for-performance targets known as the Quality and Outcomes Framework (QOF) [10]. Approximately 8% of the QOF points (worth around £10,000) per year per practice are related to the recording of smoking status and delivery of smoking cessation advice [11], [12]. GPs are required to document patients' smoking status at least once every 27 months, or every 15 months where the patient has hypertension, diabetes, asthma and certain other smoking-related morbidities. A detailed description of QOF targets is available elsewhere [13].

In the population as a whole the recording of patients' smoking status is more complete after the introduction of QOF [14], [15]. However, the QOF sets no specific incentives for the recording of smoking status in pregnant women. Having smoking status recorded in a pregnant women's medical records is not only useful for clinical management, but also increases opportunities for health professionals to provide smoking cessation interventions throughout pregnancy and afterwards. Therefore, our primary aim was to assess the completeness of recording of smoking status during pregnancy in primary care medical records over time and investigate whether completeness varied with women's sociodemographic and health-related characteristics. Additionally, our secondary aim was to investigate whether, despite having no specific targets for pregnancy, there was an increase in the completeness of smoking status recording during pregnancy in UK primary care after the introduction of the QOF.

## Methods

### Data source and study population

The Health Improvement Network (THIN) is an electronic primary care database containing anonymised patient records from general practices across the UK, covering approximately 5.7% of the UK population [16]. The version of THIN used for this study contains data from 495 practices with a combined total of approximately 9.5 million patients, including approximately 2 million women of reproductive age (defined here as age 15–49 years) [16]. The recorded population prevalence of smoking in THIN has been previously validated at both national and regional levels [14], [15] and fertility rates in THIN are highly comparable to national fertility rates [17]. For the work reported here, our study population included all pregnancies recorded in THIN between 2000 and 2009 in women of reproductive age which resulted in either a live birth or a stillbirth.

### Smoking status and maternal characteristics

Records of maternal smoking status during pregnancy were identified using Read codes [18]. These included codes for current, never, and ex-smoking, codes indicating the type or number of cigarettes smoked, and codes indicating smoking cessation interventions delivered to patients. Women were also considered to be smokers if they had a prescription for a smoking cessation drug (nicotine replacement therapy, bupropion or varenicline) in their medical records during pregnancy. This method of classifying smoking status in electronic primary care data has been previously validated [14]. Code lists are available from the authors on request.

To investigate the factors that may be associated with the recording of maternal smoking status during pregnancy, data were extracted on women's age at conception, socioeconomic deprivation as measured by quintiles of the Townsend Index of material deprivation [19], body mass index (BMI) before conception and recorded diagnoses, during or before the pregnancy, of morbidities common in pregnancy in which the recording of smoking status has been specifically incentivised by the QOF (hypertension, diabetes, asthma, and mental illness which included depression, anxiety, bipolar disorder, schizophrenia and other psychoses). When extracting data on BMI and maternal morbidities before pregnancy, we only considered recent recordings of BMI and comorbidities before pregnancy (27 months prior to pregnancy, in line with the QOF recording rules). Missing data for Townsend quintile and BMI were included as separate categories in the analyses.

### Statistical analyses

The prevalence of smoking status recording during pregnancy was calculated for each year from 2000 to 2009 as the number of pregnancies with at least one recording of smoking status during the gestational period divided by the total number of pregnancies delivered in that year. These data were plotted graphically.

Since April 2006 the QOF has not required GPs to record the smoking status of patients after the age of 25 years if they have been a never smoker until that age [20]. After 2008, if a patient who once smoked has been recorded as an ex-smoker for three years, GPs need no longer check and update the patient's smoking status records. Therefore, we recalculated the proportion of pregnancies with missing gestational smoking status data to take these rules into account. For women who only had records of being a never smoker up to age 25 and who did not have a record of smoking during a subsequent pregnancy we imputed a never smoking record during gestation. Similarly, for women who had no smoking status records during gestation but who were recorded as ex-smokers for three consecutive years before the conception we imputed an ex-smoking record during gestation. We then recalculated the annual proportion of pregnancies with a recording of smoking status during the gestational period.

We used logistic regression to calculate odds ratios (ORs) for associations between women's characteristics and the recording of smoking status during pregnancy. All covariates that reached statistical significance (p<0.05) in the univariable analysis were initially included in the multivariable analyses. Covariates that reached statistical significance (p<0.05) in the multivariable analysis were retained in the final model. As some women had more than one pregnancy during the study period that contributed to our analyses, we accounted for this potential clustering of pregnancies within women by calculating robust confidence intervals (CIs) around our odds ratios using the clustered sandwich estimator to allow for intragroup correlation [21], [22]. As the introduction of the QOF incentivised the recording of smoking status in patients with smoking-related chronic conditions, we expected the QOF to be an effect modifier of the association between recording of smoking status during pregnancy and these morbidities. We therefore carried out logistic regression for two separate time periods: before the implementation of the QOF (January 2000–April 2004) and after the implementation of the QOF (April 2004–December 2009). We visually compared the magnitude, precision and significance of the odds ratios for each maternal factor in the pre and post-QOF periods in order to assess whether the association between maternal factors and the recording of smoking status during pregnancy changed after the QOF was introduced. All analyses were performed using Stata version 12.0 (StataCorp LP, College Station, TX).

### Ethics Statement

Ethical approval for use of the THIN data was provided by the THIN Scientific Review Committee (reference number 11-047).

## Results

### Baseline characteristics

We identified 215,703 women with pregnancies resulting in live births or stillbirths between January 2000 and December 2009. Of these, 162,295 (75.0%) women had only one pregnancy, 46,062 (21.5%) had two pregnancies and 7,346 (3.5%) had three or more pregnancies, giving a total of 277,552 pregnancies. The mean age at conception was 29.5 years (standard deviation 5.9) and the average length of pregnancy was 39.4 weeks (standard deviation 2.2). Table 1 describes the baseline characteristics of the study population in the pre-QOF and post-QOF time periods. The overall prevalence of diagnosed asthma, diabetes, hypertension and mental illness within the study population was approximately 8%, 2%, 2.5% and 9% respectively. Information on socioeconomic status was missing for 6% of the total pregnancies and information on BMI was missing for 42% of pregnancies.

**Table 1:** Baseline characteristics of the study population.

	Pre-QOF(January 2000– March 2004)			Post-QOF (April 2004– December 2009)		
	Total pregnancies (n = 98,373)	Pregnancies with a gestational smoking record (n = 12,381)		Total pregnancies (n = 179,179)	Pregnancies with a gestational smoking record (n = 64,188)	
**Age at Conception**						
15–19 years	5,529	953	(17.2%)	9,854	4,856	(14.8%)
20–24 years	14,809	2,202	(14.9%)	29,323	12,607	(14.9%)
25–29 years	25,732	3,175	(12.3%)	45,416	16,758	(15.7%)
30–34 years	32,621	3,750	(11.5%)	54,574	17,437	(17.4%)
35–39 years	16,614	1,944	(11.7%)	32,778	10,296	(9.9%)
40–44 years	2,907	338	(11.6%)	6,868	2,123	(19.8%)
45–49 years	161	19	(11.8%)	366	111	(15.9%)
**Townsend Score in quintiles**						(14.4%)
Quintile 1 - most affluent	24,760	2,850	(11.5%)	38,815	11,733	(16.5%)
Quintile 2	19,288	2,277	(11.8%)	32,962	11,025	(14.8%)
Quintile 3	18,592	2,317	(12.5%)	35,209	12,542	(14.9%)
Quintile 4	17,128	2,279	(13.3%)	33,982	13,114	(15.7%)
Quintile 5 - most deprived	13,252	1,964	(14.8%)	25,742	10,915	(17.4%)
Missing	5,353	694	(13.0%)	12,469	4,859	(9.9%)
**Pre-conception Body Mass Index**						(19.8%)
Normal(18.0–24.9)	26,663	3,948	(14.8%)	59,267	21,209	(15.9%)
Underweight(<18.0)	1,968	293	(14.9%)	4,355	1,714	(14.4%)
Overweight(25–29.9)	11,923	1,867	(15.7%)	29,476	10,957	(16.5%)
Obese(> = 30)	7,125	1,240	(17.4%)	20,993	8,406	(14.8%)
Missing	50,694	5,033	(9.9%)	65,088	21,902	(14.9%)
**Asthma**	6,537	1,297	(19.8%)	16,807	8,911	(15.7%)
**Hypertension**	2,372	377	(15.9%)	4,962	1,959	(17.4%)
**Diabetes**	1,345	194	(14.4%)	4,864	1,857	(9.9%)
**Mental illness**	8,717	1,439	(16.5%)	17,294	7,373	(19.8%)

### Completeness of maternal smoking records

**Figure 1:**
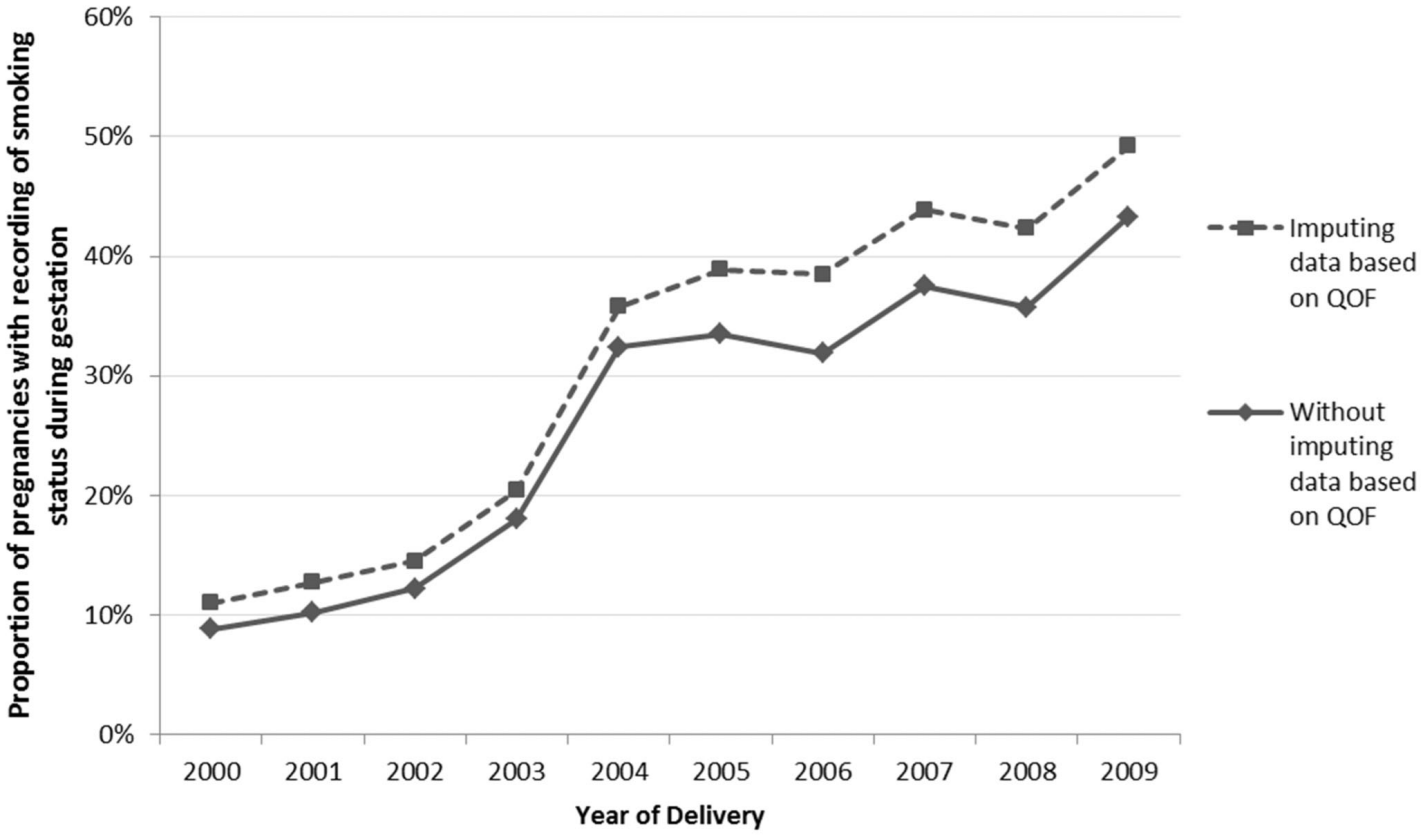
Annual proportion of pregnancies in THIN with smoking status recorded during gestation (2000–2009).

A record of smoking status at any point during the gestational period was present in 76,569 (28%) of the 277,552 pregnancies. Of the 76,569 pregnancies in which smoking status was recorded, 913 (1.2%) only had a recording for smoking cessation drug prescription with no accompanying Read codes indicating smoking status. In 56,605 (20.4%) pregnancies, women had their smoking status recorded only once during the gestational period, whereas in 19,964 (7%) pregnancies smoking status was recorded more than once. Figure 1 shows the proportion of pregnancies with smoking status recorded during gestation from 2000 to 2009. In 2000, smoking status was recorded during the gestational period for only 1,943 (8.8%) of the total 22,111 pregnancies. This proportion increased steadily to 18% in 2003 and a steep point change was observed in 2004 with the proportion rising to 32.3%. After 2004 it increased steadily on an annual basis such that the proportion of pregnancies with smoking status recorded during gestation in 2009 was 43.3% (13,360 out of 30,880 pregnancies).

When data for never smoking and ex-smoking were imputed based on QOF rules, the overall proportion of pregnancies with a record of smoking status during gestation increased to 32.1%. In 2000, smoking status was recorded during gestation for only 11.0% of pregnancies which increased to 35.8% in 2004 and 49.2% in 2009 (Figure 1).

### Factors associated with recording of maternal smoking status during pregnancy

Table 2 shows variations in the recording of smoking status during pregnancy by women's sociodemographic characteristics and morbidities in the pre-QOF and post-QOF time periods. Overall, the strength of the associations between all maternal characteristics and recording of smoking status during gestation was higher in the post-QOF period compared to pre-QOF period. The recording of smoking status during pregnancy varied with socioeconomic status such that pregnant women from the most deprived group (quintile 5) were 17% more likely to have their smoking status recorded during pregnancy than pregnant women from the most affluent group (quintile 1) before the implementation of the QOF (OR 1.17, 95% CI 1.10–1.25) and 42% more likely afterwards (OR 1.42, 95% CI 1.37–1.47). Similarly, pre-QOF pregnant women with a diagnosis of asthma were 63% more likely to have their smoking status recorded during pregnancy than pregnant women without asthma (OR 1.63, 95% CI 1.53–1.74) and post-QOF pregnant women with asthma were over twice as likely to have their smoking status recorded during pregnancy (OR 2.08, 95% CI 2.02–2.15). Having a diagnosis of diabetes was not associated with the recording of gestational smoking status pre-QOF (unadjusted OR 1.17, 95% CI 1.00–1.36), (p = 0.290). However, post-QOF it was associated with a 12% increase in the odds of recording of gestational smoking status (OR 1.12, 95% CI 1.05–1.19). Recording of smoking status during pregnancy was also related to hypertension and mental illness. In both time periods the odds of a woman having her smoking status recorded during pregnancy were greater at younger ages compared with older ages and great in overweight and obese women. However, the magnitude of effects and corresponding CIs in the pre-QOF and post-QOF periods overlapped.

**Table 2:** Odds of having smoking status recorded during gestation by women's characteristics before and after the QOF implementation.

	Pre-QOF(January 2000–March 2004)				Post-QOF(April 2004–December 2009)			
	Unadjusted		Adjusted		Unadjusted		Adjusted	
	OR (95% CI)	p-value	OR (95% CI)	p-value	OR (95% CI)	p-value	OR (95% CI)	p-value
**Age**								
15–19	1.48 (1.37–1.60)		1.56 (1.44–1.70)		1.66 (1.59–1.74)		1.62 (1.54–1.69)	
20–24	1.24 (1.17–1.32)		1.22 (1.15–1.30)		1.29 (1.25–1.32)		1.24 (1.20–1.28)	
25–29	1	<0.001	1	<0.001	1	<0.001	1	<0.001
30–34	0.92 (0.87–0.97)		0.95 (0.91–1.00)		0.80 (0.78–0.82)		0.84 (0.82–0.86)	
35–39	0.94 (0.88–0.99)		0.99 (0.93–1.05)		0.78 (0.75–0.80)		0.83 (0.80–0.85)	
40–44	0.93 (0.83–1.05)		0.99 (0.88–1.12)		0.76 (0.72–0.81)		0.80 (0.76–0.85)	
45–49	0.95 (0.59–1.53)		0.99 (0.61–1.60)		0.74 (0.59–0.93)		0.77 (0.61–0.97)	
**Townsend Score**								
Quintile 1 (most affluent)	1		1		1		1	
Quintile 2	1.03 (0.78–1.09)		1.01 (0.95–1.07)		1.16 (1.12–1.19)		1.12 (1.09–1.16)	
Quintile 3	1.09 (1.03–1.16)	<0.001*	1.03 (0.97–1.10)	<0.001*	1.28 (1.24–1.32)	<0.001*	1.18 (1.14–1.21)	<0.001*
Quintile 4	1.18 (1.11–1.25)		1.07 (1.00–1.13)		1.45 (1.40–1.49)		1.26 (1.22–1.30)	
Quintile 5 (most deprived)	1.34 (1.25–1.42)		1.17 (1.10–1.25)		1.69 (1.64–1.75)		1.42 (1.37–1.47)	
Missing	1.14 (1.04–1.25)		1.06 (0.97–1.16)		1.47 (1.41–1.54)		1.34 (1.29–1.40)	
**Body Mass Index**								
Underweight (<18.0)	1.01(0.88–1.14)		0.92 (0.81–1.05)		1.16 (1.10–1.24)		1.03 (0.97–1.10)	
Normal (18.0–24.9)	1		1		1		1	
Overweight (25.0–29.9)	1.07 (1.01–1.13)	<0.001	1.06 (1.00–1.13)	<0.001	1.06 (1.03–1.09)	<0.001	1.05 (1.02–1.09)	<0.001
Obese (≥30)	1.21 (1.13–1.30)		1.16 (1.08–1.25)		1.19 (1.16–1.23)		1.11 (1.08–1.15)	
Missing	0.63 (0.60–0.66)		0.63 (0.60–0.66)		0.91 (0.89–0.93)		0.90 (0.88–0.92)	
**Asthma**	1.80 (1.69–1.92)	<0.001	1.63 (1.53–1.74)	<0.001	2.19 (2.12–2.25)	<0.001	2.08 (2.02–2.15)	<0.001
**Hypertension**	1.32 (1.18–1.48)	<0.001	1.26 (1.12–1.41)	<0.001	1.17 (1.11–1.24)	<0.001	1.19 (1.12–1.26)	<0.001
**Diabetes**	1.17 (1.00–1.36)	0.045	- ‡	-‡	1.11 (1.05–1.18)	<0.001	1.12 (1.05–1.19)	<0.001
**Mental illness**	1.42 (1.34–1.51)	<0.001	1.32 (1.24–1.41)	<0.001	1.37 (1.33–1.41)	<0.001	1.26 (1.22–1.30)	<0.001

OR = odds ratio, CI = confidence interval, QOF = Quality and Outcomes Framework,

* p-value for trend,

‡ Diabetes not significant in the final model.

## Discussion

Using a large population-based dataset we found that the recording of smoking status during pregnancy in primary care has improved with time such that the proportion of pregnancies with a recording of smoking status during gestation was 8.8% in 2000 rising to 43.3% in 2009. The odds of a woman's smoking status being recorded during pregnancy was related to age, socioeconomic deprivation, BMI and QOF-incentivised morbidities such as asthma, diabetes, hypertension and mental illness.

The proportion of pregnancies with a gestational smoking record increased by approximately 2% per year between 2000 and 2002. Since the late 1990s there has been an increased focus on the harms of tobacco use in the UK, with, for example, the publication of the Government white paper ‘Smoking Kills’ in 1998 [1], the establishment of NHS Stop Smoking Services in 1999 [23], and the availability of smoking cessation medications on NHS prescriptions from 2001 [24]. This changing tobacco control environment may have made these pregnant smokers more willing to approach their GPs for help to quit, and focused GPs' attention on encouraging cessation in their patients, thereby increasing the proportion of pregnant women with a smoking status record in their medical notes. The proportion of pregnancies with a recording of smoking status rose sharply from 18.0% in 2003 to 32.4% in 2004, after which it increased slowly until 2009. The most plausible explanation for this marked increase between 2003 and 2004 is GPs' awareness of the impending introduction of the 2004 GP contract [25]. Similar improvements in the recording of smoking status have been seen in general population. A study using primary care data for over 300 practices throughout the UK found that, although rates of recording of smoking status in patients' electronic medical records had been increasing gradually since the year 2000, the rate of improvement accelerated from 2003, with an 88% increased observed between the first quarter of 2003 and the same period in 2004, just before the introduction of the QOF [26]. This suggests that the introduction of the QOF resulted in better recording of smoking status in the general population which has spilled over into the greater recording in pregnancy observed in our study.

For socioeconomic deprivation, asthma and diabetes the magnitude of effect of the association with smoking status recording was observed to be stronger after the introduction of the QOF. Pre-QOF, pregnant women from the most deprived group were 17% more likely to have their smoking status recorded during gestation compared to 42% post-QOF. Smoking prevalence is generally higher in lower socioeconomic groups in both the general population as well as amongst pregnant women [27] and the smoking status of smokers is more likely to be recorded than that of non-smokers [28]–[30], which likely explains more complete recording in pregnant women from lower socioeconomic groups. Furthermore, low socioeconomic status is associated with a higher prevalence of chronic diseases such as hypertension, diabetes, asthma and depression [31]. The QOF encourages improved clinical management of these patients, who post-QOF may have had more frequent contacts with their GP and thus have had more chance of being asked about their smoking behaviour, increasing the gradient of the association between socioeconomic status and smoking status recording, reflecting that recording, and thus hopefully monitoring, is more complete where it is most needed [32], [33]. Asthma is the most common pre-existing condition encountered during pregnancy [34] and is closely related to smoking, which may explain the high magnitude of association between asthma and recording of smoking status compared to other conditions like diabetes (which affects approximately 2–5% in women of reproductive age) [35] and hypertension (0.6–2.7% during pregnancy) [36]. Women with a higher BMI have an increased risk of complications during pregnancy and therefore are more likely to visit their GPs [37]. They are also more likely to be smokers which in turn will affect the completeness of recording of their smoking status. Our findings are similar to those from a study in the general population which found that primary care patients with smoking-related chronic medical conditions and greater social deprivation were more likely to have a recent recording of smoking status or cessation advice in their medical records [38]. However, the magnitude of effect in this general population study for all morbidities much higher than that which we found, presumably because currently pregnancy is not a QOF-incentivised condition for recording of smoking.

To our knowledge this is the first study to assess the completeness of recording of smoking status during pregnancy in UK primary care medical records at a national level, using a large population-based dataset with over 200,000 pregnancies. A potential limitation of our study is that due to the infrequency of smoking status recordings during pregnancy we did not assess recording of smoking status in smaller windows during pregnancy such as in each trimester, which may be more appropriate given that smoking status fluctuates during pregnancy [39]. Furthermore, we only assessed electronically-coded data in primary care records to examine the recording of smoking status during pregnancy and did not have access to free text or midwives' notes to ascertain smoking status; these may provide additional information on the smoking status of women during pregnancy. A potential explanation for the high proportion of pregnancies in which smoking status was not recorded could be that if a woman's smoking habit did not change after she became pregnant, GPs might be less likely to re-enter this information into medical records as there is no specific financial incentive for recording smoking status in pregnant women. Furthermore, as the QOF does not require GPs to record the smoking status of ‘never smokers’ after the age of 25, there is no financial incentive for them to update smoking status in the medical records of women who have never smoked. Similarly, ex-smokers need only be asked about their smoking status annually until they have been a non-smoker for three years. However, when we recalculated smoking status based on these rules, the annual trends in the completeness of smoking data during pregnancy did not vary much from the trends using the original data.

The current antenatal model in the UK is a midwife-led care one , where midwives are the main point of contact for women during pregnancy [9], [40]. The National Institute for Health and Clinical Excellence (NICE) recommends that all pregnant women should have their smoking status recorded at the first antenatal booking appointment with the midwife and that all smokers should be referred to a stop smoking service and this should be recorded in the hand-held records which women in the UK carry with them throughout their pregnancy [7], [41]. Data from a qualitative study of midwives in Glasgow, Scotland, suggested that they view it as part of their role to collect this smoking data at the booking appointment [42]. However, the means of recording of maternity data and provision of smoking cessation information during antenatal visits varies from practice to practice and we do not know whether, or how completely, smoking status data entered onto hand-held records get transferred to a woman's electronic primary care medical record for future reference.

As the current guidelines recommend, monitoring of smoking status during pregnancy should be a shared responsibility between all healthcare professionals involved in the care of pregnant women, including GPs and midwives [4], [5], [7]. The Royal College of Midwives recommends that during pregnancy midwives should have full confidential access to a woman's written and electronic records and GPs should ensure that all significant and relevant information is copied into a woman's hand-held maternity records [8]. Similarly, relevant information collected by midwives during pregnancy should also be communicated to the GPs and fed back into the electronic primary care records. Therefore, we recommend that appropriate methods should be introduced to improve communication and documentation of such information between the midwives and the GPs during pregnancy. One such strategy could be inclusion of pregnancy in the QOF as a condition where smoking status and smoking cessation advice should be recorded in the electronic primary care record. Primary care is the central hub in the current UK health care system and increasing the assessment and complete documentation of smoking status in primary care will not only increase opportunities for providing smoking cessation advice and interventions during pregnancy, but is also important to maintain continuity of care throughout and beyond pregnancy for both a woman's health and that of her children.

## References

[1] Department of Health (1998) Smoking kills. Great Britain: Stationary Office.

[2] McCowan LME, Dekker GA, Chan E, Stewart A, Chappell LC, et al.. (2009) Spontaneous preterm birth and small for gestational age infants in women who stop smoking early in pregnancy: prospective cohort study. Br Med J. 338. doi:10.1136/bmj.b1081 10.1136/bmj.b1081 PMC2661373 19325177

[3] Lindley AA, Becker S, Gray RH, Herman AA (2000) Effect of continuing or stopping smoking during pregnancy in infant birth weight, crown-heel length, head circumference, ponderal index, and brain∶body weight ratio. Am J Epidemiol 152: 219–225. 10933268 10.1093/aje/152.3.219

[4] National Institute for Health and Clinical Excellence (2010) PH26 Quitting smoking in pregnancy and following childbirth: guidance. Available: http://guidance.nice.org.uk/PH26/Guidance/pdf/English. Accessed 2012 May 16.

[5] National Institute for Health and Clinical Excellence (2008) Smoking cessation services in primary care, pharmacies, local authorities and workplaces, particularly for manual working groups, pregnant women and hard to reach communities. London: National Institue for Health and Clinical Excellence.

[6] National Institute for Health and Clinical Excellence (2010) Antenatal care. London: National Institue for Health and Clinical Excellence.

[7] National Institute for Health and Clinical Excellence (2012) Quality standard for antenatal care. Quality statement 5: Risk assessment - smoking cessation. Manchester: National Institute for Health and Clinical Excellence

[8] Royal College of Obstetricians and Gynaecologists (2008) Standards for Maternity Care - Report of a Working Party. London: RCOG Press.

[9] Redshaw M, Heikkila K (2010) Delivered with care: a national survey of women's experience of maternity care in 2010. Oxford: National Perinatal Epidemiology Unit - University of Oxford.

[10] The Information Centre The Quality and Outcomes Framework. Available: http://www.ic.nhs.uk/statistics-and-data-collections/audits-and-performance/the-quality-and-outcomes-framework. Accessed 2012 Sep 9.

[11] Simon C (2008) The Quality and Outcomes Framework. InnovAiT 1: 206–213.

[12] Ward P (2007) Scoring top marks for smoking cessation. The British Journal of Primary Care Nursing 1: 129–131.

[13] Primary Care Commissioning (2013) Available: http://www.pcc-cic.org.uk/general-practice/contracts. Accessed 2013 May 26.

[14] Szatkowski L, Lewis S, McNeill A, Huang Y, Coleman T (2011) Can data from primary care medical records be used to monitor national smoking prevalence? J Epidemiol Community Health doi:10.1136/jech.2010.120154 10.1136/jech.2010.120154 21571750

[15] Langley TE, Szatkowski L, Wythe S, Lewis S (2011) Can primary care data be used to monitor regional smoking prevalence? An analysis of The Health Improvement Network primary care data. BMC Public Health 11. 10.1186/1471-2458-11-773 PMC3198710 21981754

[16] CSD Medical Research UK (2011) THIN Data Guide for Researchers.

[17] Tata LJ, Hubbard RB, McKeever TM, Smith CJP, Doyle P, et al (2007) Fertility rates in women with asthma, eczema, and hay fever: a general population-based cohort study. Am J Epidemiol 165: 1023–1030. 17255115 10.1093/aje/kwk092

[18] NHS Connecting for Health Read Codes. Available: http://www.connectingforhealth.nhs.uk/systemsandservices/data/uktc/readcodes. Accessed 2012 Jan 15.

[19] Townsend P, Phillimore P, Beattie A (1988) Health and deprivation: Inequality and the North. London: Croom Helm.

[20] National Health Service Information Centre - QOF Business Rules team (2011) New GMS contract QOF implementation dataset and business rules - Smoking indicator set. London: Department of Health.

[21] StataCorp. (2009) Stata: Release 11. Statistical Software. College Station, TX: StataCorp LP.

[22] Williams RL (2000) A note on robust variance estimation for cluster-correlated data. Biometrics 56: 645–646. 10877330 10.1111/j.0006-341x.2000.00645.x

[23] The Information Centre (2007) Statistics on NHS Stop Smoking Services in England, April to September 2006. Available: https://catalogue.ic.nhs.uk/publications/public-health/smoking/nhs-stop-smok-serv-eng-2006-q2/nhs-stop-smok-serv-eng-2006-q2-rep.pdf. Accessed 2013 May 26.

[24] Langley TE, Huang Y, Mcneill A, Coleman T, Szatkowski L, et al (2011) Prescribing of smoking cessation medication in England since the introduction of varenicline. Addiction 106: 1319–1324. 21395894 10.1111/j.1360-0443.2011.03426.x

[25] Gillam S, Siriwardena AN (2011) The Quality and Outcomes Framework QOF - transforming general practice. Abingdon: Radcliffe Publishing Ltd.

[26] Coleman T, Lewis S, Hubbard R, Smith C (2007) Impact of contractual financial incentives on the ascertainment and management of smoking in primary care. Addiction 102: 803–808. 17506157 10.1111/j.1360-0443.2007.01766.x

[27] The NHS Information Centre IFF Research (2011) The infant feeding survey 2010: early results. York: The NHS Information Centre.

[28] Mant D, Murphy M, Rose P, Vessey M (2000) The accuracy of general practitioner records of smoking and alcohol use: comparison with patient questionnaires. J Public Health 22: 198–201. 10.1093/pubmed/22.2.198 10912559

[29] Mant D, Phillips A (1986) Can the prevalence of disease risk factors be assessed from general practice records. Br Med J 292: 102–103. 3080076 10.1136/bmj.292.6513.102 PMC1339118

[30] Wilson A, Manku-Scott T, Shepherd D, Jones B (2000) A comparison of individual and population smoking data from a postal survey and general practice records. Br J Gen Pract 50: 465–468. 10962784 PMC1313724

[31] Eachus J, Williams M, Chan P, Smith GD, Grainge M, et al (1996) Deprivation and cause specific morbidity: evidence from the Somerset and Avon survey of health. Br Med J 312: 287–292. 8611787 10.1136/bmj.312.7026.287 PMC2349904

[32] Julian TH (1971) The inverse care law. The Lancet 297: 405–412. 10.1016/s0140-6736(71)92410-x 4100731

[33] Watt G (2002) The inverse care law today. The Lancet 360: 252–254. 10.1016/S0140-6736(02)09466-7 12133675

[34] Nelson-Piercy C (2001) Asthma in pregnancy. Br Med J 56: 325–328. 10.1136/thorax.56.4.325 PMC1746013 11254828

[35] National Collaboring Centre for Women's and Children's Health (2008) Diabetes in pregnancy - Management of diabetes and its complications from pre-conception to the postnatal period. London: National Institute for Health and Clinical Excellence.

[36] National Collaboring Centre for Women's and Children's Health (2011) Hypertension in pregnancy - the management of hypertensive disorders during pregnancy. London: National Institute for Health and Clinical Excellence.

[37] National Institute for Health and Clinical Excellence (2010) Weight management before, during and after pregnancy. Manchester: National Institute for Health and Clinical Excellence.

[38] Taggar JS, Coleman T, Lewis S, Szatkowski L (2012) The impact of the Quality and Outcomes Framework (QOF) on the recording of smoking targets in primary care medical records: cross-sectional analyses from the Health Improvement Network (THIN) database. BMC Public Health 12: 329–339. 22559290 10.1186/1471-2458-12-329 PMC4104830

[39] Pickett KE, Wakschlag LS, Dai L, Leventhal BL (2003) Fluctuations of maternal smoking during pregnancy. Obstet Gynecol 101: 140–147. 12517659 10.1016/s0029-7844(02)02370-0

[40] Smith A, Shakespeare J, Dixon A (2010) The role of GPs in maternity care- what does the future hold? London: The King's Fund.

[41] National Institute for Health and Clinical Excellence (2012) Referral pathway for pregnant women who smoke. London: National Institute for Health and Clinical Excellence.

[42] Bauld L, Wilson M, Kearns A, Reid M (2007) Exploring reductions in smoking during pregnancy in Glasgow. Glasgow: University of Glasgow, University of Bath.

